# Global rank-invariant set normalization (GRSN) to reduce systematic distortions in microarray data

**DOI:** 10.1186/1471-2105-9-520

**Published:** 2008-12-04

**Authors:** Carl R Pelz, Molly Kulesz-Martin, Grover Bagby, Rosalie C Sears

**Affiliations:** 1 Department of Molecular and Medical Genetics, Oregon Health and Sciences University, Portland, OR 97239-3098, USA; 2 Department of Dermatology, Oregon Health and Sciences University, Portland, OR 97239-3098, USA; 3 Department of Cell and Developmental Biology, Oregon Health and Sciences University, Portland, OR 97239-3098, USA; 4 Department of Medicine, Oregon Health and Sciences University, Portland, OR 97239-3098, USA; 5 OHSU Knight Cancer Institute, Oregon Health and Sciences University, Portland, OR 97239-3098, USA

## Abstract

**Background:**

Microarray technology has become very popular for globally evaluating gene expression in biological samples. However, non-linear variation associated with the technology can make data interpretation unreliable. Therefore, methods to correct this kind of technical variation are critical. Here we consider a method to reduce this type of variation applied after three common procedures for processing microarray data: MAS 5.0, RMA, and dChip^®^.

**Results:**

We commonly observe intensity-dependent technical variation between samples in a single microarray experiment. This is most common when MAS 5.0 is used to process probe level data, but we also see this type of technical variation with RMA and dChip^® ^processed data. Datasets with unbalanced numbers of up and down regulated genes seem to be particularly susceptible to this type of intensity-dependent technical variation. Unbalanced gene regulation is common when studying cancer samples or genetically manipulated animal models and preservation of this biologically relevant information, while removing technical variation has not been well addressed in the literature. We propose a method based on using rank-invariant, endogenous transcripts as reference points for normalization (GRSN). While the use of rank-invariant transcripts has been described previously, we have added to this concept by the creation of a global rank-invariant set of transcripts used to generate a robust average reference that is used to normalize all samples within a dataset. The global rank-invariant set is selected in an iterative manner so as to preserve unbalanced gene expression. Moreover, our method works well as an overlay that can be applied to data already processed with other probe set summary methods. We demonstrate that this additional normalization step at the "probe set level" effectively corrects a specific type of technical variation that often distorts samples in datasets.

**Conclusion:**

We have developed a simple post-processing tool to help detect and correct non-linear technical variation in microarray data and demonstrate how it can reduce technical variation and improve the results of downstream statistical gene selection and pathway identification methods.

## Background

Given the large volume of data generated by microarray technology and the many sources of variation involved, including not only biologically relevant variation, but also technical variation that results from sample preparation and labeling, hybridization, and other processing steps [1], methods for analyzing and interpreting the results are very important. An important component of these technologies is redundancy. For example, the Affiymetrix^® ^GeneChip^® ^platform, employs many features (probes) to interrogate each gene (transcript). These redundant probes are called a "probe set" and summarizing each probe set to arrive at a robust value representing the abundance of the associated transcript is one of the first steps in any analysis [2]. Another important step is normalization to remove systematic array to array variation such as differential hybridization and scanning artifacts. Some popular processing methods for these steps are the MAS 5.0 algorithm [3] developed by Affymetrix^®^, the Robust Multichip Average (RMA) method developed by Irizarry et al. [2], and the dChip^® ^method developed by Li et al. [4]. Each of these include normalization methods: MAS 5.0 uses a simple global scaling factor [3], RMA uses quantile normalization [5], and dChip^® ^ uses a rank-invariant set based method [4]. By definition, the global scaling method of MAS 5.0 cannot handle non-linear artifacts. Furthermore, it may not even produce the optimal linear scaling factor as suggested by Lu [6]. Still, the MAS 5.0 method is commonly used and even preferred by some researchers for some applications [7]. RMA has a number of advantages over MAS 5.0 including quantile normalization, which is a mathematically elegant solution for setting the intensity distributions equal for all arrays in the dataset [2,5,8]. However this is not always appropriate and may cause problems when the assumption of equal distributions is not met, for example when more probe sets are up-regulated than down-regulated as discussed by Freudenberg et al. [9]. dChip^® ^uses a rank-invariant set based normalization method on probe level data, however the dChip^® ^approach selects one reference sample and compares all other samples to it, selecting a different rank-invariant set to normalize each sample. Using our proposed method based on a global rank-invariant set to create a robust average reference, which we refer to as Global Rank-invariant Set Normalization (GRSN) and applying it to the summarized probe set data from dChip^®^, we see a further reduction in specific types of systematic array to array variation.

For the purpose of this discussion, we will classify the variation of microarray data into three categories: biological, random, and systematic. Biological variation is of interest to the researcher and may contain many different components. The random and systematic categories are both forms of technical variation. Random variation has no biological relevance and is the result of uncharacterizable measurement errors. Systematic variation also has no biological relevance, but is characterizable as a function of expression value. We are interested in systematic variation because it can be "modeled" and removed. For example, if any of the microarray processing steps (labeling, hybridization, scanning, etc.) are non-linear functions of transcript abundance, and the conditions affecting these non-linear functions change from array to array or with biologic condition due to unbalanced gene expression, then systematic variation will be introduced between arrays and/or conditions. The goal of this work is to graphically show and mathematically remove this type of variation, which we refer to as "non-linear artifacts" or "skew". We use rank-invariant transcripts as reference points to detect and remove these non-linear artifacts. This is not a new concept, for example, Li and Wong [4] use rank-invariant probes for normalization in their dChip software. However, we have extended this idea by selecting a global set of endogenous, rank-invariant, transcripts to generate average reference points used to normalize all samples in a dataset. We also apply our normalization as an additional step after using existing methods for summarizing probe sets. The efficacy of additional normalization at the "probe set level" has been advocated by others [10]. Using a global rank-invariant set (as opposed to selecting a new rank-invariant set for each sample) reduces the risk of introducing noise into the dataset. Applying our method as a post probe set summary method allows us the flexibility of using our favorite probe set summary method. In addition, the summarized probe set values should give the best estimate of true gene expression, so it makes sense to use these values when selecting the rank-invariant set. With the use of optimized probe set definitions as described by Sandberg and Larson [11], this may become even more beneficial.

In our experience with microarray datasets, both from human cancer studies and animal models, it is common to have unbalanced up or down regulation of gene expression between two sample populations. When using the standard data processing methods discussed above, we often see an intensity-dependent skew when comparing conditions in such data. This skew, in turn, introduces errors in further statistical analysis and in the calculation of fold change. These errors will bias the results of gene selection based on statistics and fold change and can lead to the detection of "statistically significant" genes that are not in fact differentially expressed. GRSN is a simple, yet robust, method for reducing this type of distortion, and minimizing the chances of obtaining misleading analysis results. We use simulated data to show that GRSN reliably reduces non-linear skew even when actual gene expression is highly unbalanced. GRSN does not introduce bias into the dataset by trying to balance the number or magnitude of up and down regulated genes. As a result, GRSN performs well on a wide range of datasets, including datasets with as few as two samples.

## Results

### Visualizing non-linear technical variation in microarray data

**Figure 1:**
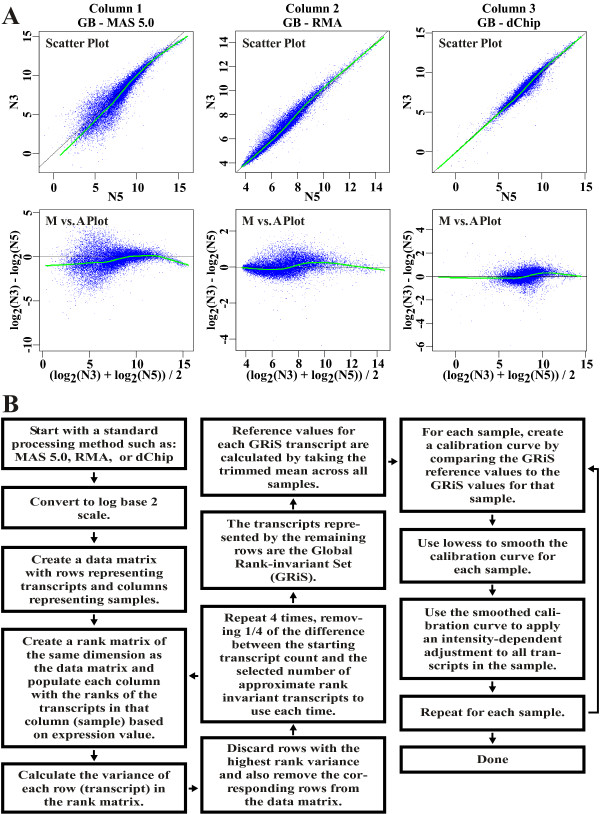
**Visualization of typical non-linear artifacts in microarray data and the GRSN method used to reduce them**. A. Visualizing non-linear technical artifacts. Top row – standard log base 2 scatter plots comparing normal sample N3 to normal sample N5 from a clinical study of Fanconi Anemia, (GB dataset). Bottom row – the same data as in the top row, but plotted using M vs. A plots in which M is plotted as a function of A where M = log2(Y) – log2(X) and A = (log2(Y) + log2(X))/2 with X = expression values for sample 1 and Y = expression values for sample 2. The probe set summary methods used are (from left to right): MAS 5.0, RMA, and dChip^®^. B. A flow chart showing the basic steps followed by the GRSN algorithm to reduce the type of non-linear artifact shown in A.

In microarray datasets, we expect only a small fraction of the transcripts interrogated by an array to be differentially expressed. Therefore, when utilizing a scatter plot to compare pairs of samples, we expect to see most transcripts centered along the diagonal line. When this is not the case, further normalization may be required. We have examined over 30 publicly available datasets, and found many to contain samples with systematic non-linear distortions apparent in their scatter plots. In this report, we will consider a variety of datasets demonstrating various degrees of non-linear distortions, and the effect of GRSN correction. An example of non-linear distortions between microarray samples within a dataset is shown in Fig. 1A. This graph compares two normal samples from a study of the inherited disease, Fanconi Anemia (GB dataset) using patient bone marrow samples run on the Affymetrix^® ^HG-U133A GeneChip^®^. There is a distinctive curve to the data in the scatter plot (top left panel) when the MAS 5.0 method is used to process the data. This "frown" is even more evident when the data is plotted using a standard M vs. A plot (bottom left panel). The M vs. A plot [12] provides an optimal visualization of the ratio of two samples as a function of expression level. In Fig. 1A, columns 2 and 3, although not as pronounced, we also see a systematic skewing of the data when the RMA or dChip methods are used to process this data (most apparent on the M vs. A style plots). Similar distortions can be seen in other samples in this dataset and additional examples from this and other datasets are shown in subsequent figures. We have developed a method called Global Rank-invariant Set Normalization (GRSN) in an effort to reduce this type of non-linear technical variation.

### Global Rank-invariant Set Normalization

GRSN is based on the general idea of rank-invariant genes presented by Li and Wong [4]. We extend this idea to select a single, globally rank-invariant set of endogenous genes to be used to normalize all samples in a dataset. These are genes believed to be consistently expressed in all samples within a given dataset and should appear in roughly the same rank order in each sample when sorted by expression level. Importantly, this ordering, or rank, should not be affected by the types of non-linear artifacts that this normalization method is designed to correct.

An overview of the GRSN method is shown in Figure 1B. Briefly, all transcripts (representing endogenous genes) are ranked in each sample of a dataset based on expression (as calculated by summarizing probe sets using established methods such as RMA or MAS 5.0). The variance of the rank order for each transcript is then calculated across all of the samples. Transcripts with the highest rank variance are discarded. The remaining transcripts are again ranked and the process is repeated in an iterative fashion. This iteration cycle is important because, for datasets with unbalanced numbers of up and down regulated transcripts, there can be a global shift of transcript rank order caused by the most differentially regulated transcripts. This global shift of the rank order will disappear as the most differentially regulated transcripts (with the highest rank variance) are discarded during the first few iteration cycles. Note that if we require the global rank-invariant set to have rank variance of zero for all transcripts, we will not typically have enough transcripts for an effective calibration curve, i.e. the set of transcripts with exactly the same rank order in all samples is too small. Therefore, the iteration cycle is terminated when a reasonable number of approximately rank-invariant transcripts remain (5000 by default). These probe sets are considered the "Global Rank-invariant Set" (GRiS).

**Figure 2:**
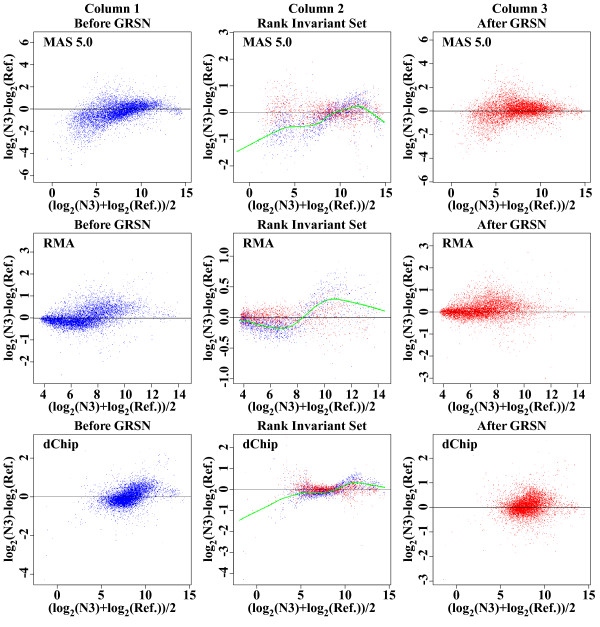
**GRSN corrects non-linear distortions apparent in different summary methods**. M vs. A plots demonstrating the GRSN method applied to the MAS 5.0, RMA, and dChip^® ^probe set summary methods. Column 1 shows M vs. A plots comparing one selected sample to the virtual reference sample created by taking the trimmed mean expression value of each probe set in that dataset. Column 2 shows the global rank-invariant set (GRiS) of 5,000 probe sets before GRSN normalization in blue and after normalization in red (note change in y-axis scale). The smoothed curve through the rank-invariant set is shown in green. This is the calibration curve used to normalize the selected sample. Column 3 shows all probe sets after GRSN normalization of the selected sample compared to the virtual reference sample. The sample shown is N3 from the GB dataset. The probe set summary methods used are (from top to bottom): MAS 5.0, RMA, and dChip^®^.

A single virtual reference sample is then created by taking the trimmed mean (mean after removing 25% of the values from the top and bottom of the range) expression value (over the entire dataset) for each summarized probe set (transcript), and M vs. A plots are generated comparing each sample to this virtual reference. This provides a visualization of the effect of applying GRSN. Fig. 2, column 1 shows the M vs. A plots comparing sample (N3) to the virtual reference of the GB dataset after the data is summarized using MAS 5.0 (first row), RMA (second row), or dChip (last row). We then generate a M vs. A plot of the identified GRiS transcripts only, comparing expression values from a given sample to expression values from the virtual reference sample (Fig. 2, column 2, blue points). We use lowess [13] to fit a smooth curve through these points (green line). This smoothed curve is used as the calibration curve for this sample. We then calculate an intensity-dependent adjustment for transcripts in each sample which, when applied, will center the sample's GRiS on the horizontal line of the M vs. A plot (at M = 0). Fig. 2, column 2, also shows the GRiS after calibration of this sample (red dots). Fig. 2, column 3, then shows all transcripts after calibration of the sample compared to the virtual reference sample (red dots). This process is repeated for each sample, with a different calibration curve generated each time (using the same GRiS). Using the trimmed mean values of the GRiS as the reference for normalization provides a robust average across all samples so that the linearity of the normalized data is not affected by a few samples with anomalous non-linear artifacts. Note that these intensity-dependent adjustments are applied additively to the log scaled data and that this is equivalent to an intensity-dependent scaling of the original, non-log scaled data.

### Determining global rank-invariant set size

**Figure 3:**
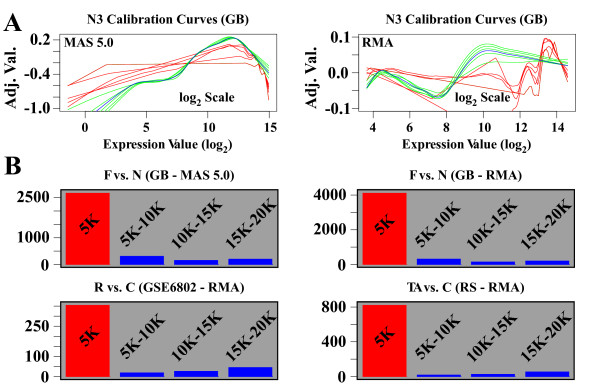
**Selection of the global rank-invariant set size**. A. The effect of selecting different sized Global Rank-invariant Sets (GRiS) on the calibration curves for a given sample. Each curve shows the GRSN calculated adjustment value as a function of expression value (for a given sample). Red curves are from GRiS sizes of 100, 200, 300, 400, and 500. Green curves are from GRiS sizes of 2000, 4000, 6000, 8000, and 10000. The blue curve is from the default GRiS size of 5000. GB dataset comparing Fanconi vs. Normal using MAS 5.0 processed data on left and RMA processed data on the right (notice different y-axis scales). B. The effect of changing the GRiS size on the selection of significantly regulated genes. Each bar represents the difference in the lists of significant genes found due to a change in the size of the GRiS. The red bar represents the default size of 5000 vs. none (not using GRSN) and is meant to show the magnitude of the effect of applying GRSN (a reference point for the other bars). The three blue bars represent, in order from left to right, the difference of 5000 to 10000, 10000 to 15000, and 15000 to 20000. Top Row – GB dataset comparing Fanconi vs. Normal using MAS 5.0 processed data on left and RMA processed data on the right. On left, GSE6802 dataset comparing R vs. C, using RMA. On right, 339RS dataset comparing TA vs. C using RMA.

When selecting the GRiS, we aim to minimize the rank order variation among transcripts in the set. We do not attempt to select a set with no rank variation because this would normally result in too few transcripts to define a smooth calibration curve. Therefore, when choosing the size of the global rank-invariant set, we must balance the desire for rank-invariant transcripts with the need for a sufficient number of calibration points. The effect of selecting too few transcripts is demonstrated in Fig. 3A. Here, multiple calibration curves (using different numbers of approximately global rank-invariant transcripts) are graphed for a single sample. The five red curves represent calibration curves generated using GRiS sizes of 100, 200, 300, 400, and 500. At this size range, the curves are erratic and segmented. The five green curves represent sizes of 2000, 4000, 6000, 8000, and 10,000. At this size range, the calibration curves smooth out and become more consistent. We conclude that GRiS sizes in the range of 100 to 500 are insufficient, but that sizes in the range of 2000 to 10,000 appear to be adequate.

Next, we look at the effect of different GRiS sizes on the detection of statistically significant genes. For each candidate rank-invariant set size, we apply the GRSN method followed by a statistical analysis to identify lists of up and down regulated genes. We used the eBayes and topTable functions from the limma [14] package in BioConductor with a FC cutoff of 1.5 and a False Discovery Rate (FDR) cutoff of 0.05 (5%) to select statistically significant genes. The FDR method [15,16] applies to multiple hypothesis testing. It uses calculated P-values to control the rate of false positives expected from a set of statistical tests. To compare two candidate sizes for the GRiS, we compare these lists of genes. For the two up regulated lists, we count the number of genes that are in one or the other list, but not both. We do the same for the down regulated lists and add the results. This gives us the number of genes affected by the change in the rank-invariant set size. In Fig. 3B, we use a bar graph to report the numbers of affected genes when different rank-invariant set sizes are compared. As a reference point, we compare GRSN with a GRiS size of 5,000 to no GRSN normalization (red bar). This serves to quantify the effect of the GRSN method itself. To quantify the "stability" at reasonably sized rank-invariant sets, we compare 5 K to 10 K, 10 K to 15 K and 15 K to 20 K (blue bars). The effect of applying GRSN (red bar) is large while the effect of changing the rank-invariant set size above 5 K (blue bars) is small. In summary, the size of the rank-invariant set does not seem to be critical. Any value in the range of 5 K to 20 K should work equally well on the current high-density arrays. However, given that we want to minimize rank variance in our selected GRiS, we use a default size of 5 K (5000) for high density arrays with greater than 20,000 probe sets.

**Figure 4:**
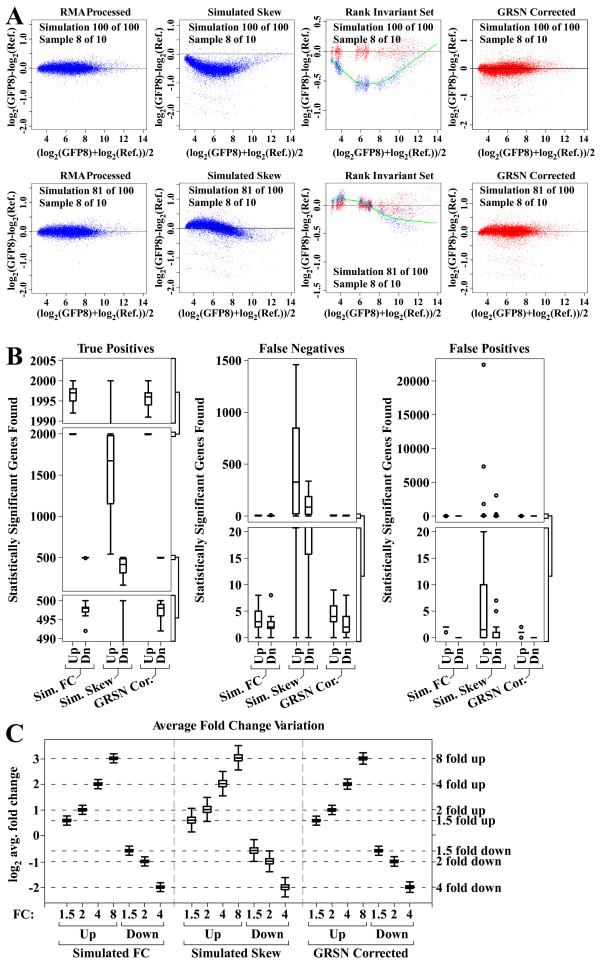
**Simulation study showing the performance of GRSN**. Differential gene expression was simulated starting with a dataset containing 10 biological replicates. The simulation was repeated 100 times and each time the 10 samples were divided into two equal groups, gene expression was simulated in the second group, and a randomly generated, non-linear artifact (skew) was applied to each sample. In each simulation, GRSN was applied to correct the simulated skew. A. First row – sample 8 of 10 from simulation 100 of 100. Left hand panel shows M vs. A plot before simulated skew. Second panel from left shows the introduction of simulated skew. Third panel from left shows GRiS and calibration curve from GRSN process. Right hand panel shows data after GRSN correction of simulated skew. Second row – same as first row, but for simulation 81 of 100. B. GRSN improves gene selection results. Left panel shows true positive gene selection results for simulated up and down genes as indicated before simulated skew is added, after simulated skew is added, and after GRSN is used to correct the simulated skew. Critical portions of the y-axis scale are expanded at the top and bottom of the graph. Middle panel shows false negatives with the bottom portion of the y-axis scale expanded at the bottom. Right panel shows false positive results. Data is represented using standard Tukey box plots. C. Average Fold Change (FC) variation. The average FC for each simulated FC value is plotted showing the variation over all simulated genes and 100 simulations. The left third of the graph shows values for simulated data before skew is introduced, the middle third shows values with skew added, and the right third shows values after GRSN correction of skew as indicated. Box plots are shown.

The choice of the smoother span supplied to the lowess function (see Methods section) can also effect the calibration curves. We have evaluated a range of values for this parameter (data not shown) and have chosen 0.25 as the default for GRSN. However, in a few cases, this default value may not be optimal. For example, some datasets (such as the simulation study presented below) produce a GRiS that is not evenly distributed along the full transcript expression range. In these cases, a larger smoother span may be needed to produce a smooth calibration curve. In the case of the simulation study described below, we chose 0.50 for the smoother span (see Fig. 4A). The tradeoff is that increasing the smoother span can lead to calibration curves that do not properly track the GRiS at the extreme ends of the transcript expression range. We recommend starting with the default value of 0.25, but checking the calibration curves plotted by the GRSN method for continuity with the GRiS (see Fig. 2, column 2).

### GRSN improves statistical performance in simulated data

When evaluating the performance of GRSN on a given microarray dataset, we are confronted with the typical problem of not knowing a priori which transcripts are truly regulated and by how much. Therefore, we have created simulated datasets where we have artificially introduced differential gene expression so that we do know a priori which genes are regulated and to what degree. We then introduce simulated, systematic, non-linear artifacts (skew) typical of what are seen in real world datasets. This data allows us to evaluate the ability of standard statistical methods to identify the correct up and down regulated genes before the simulated artifacts are introduced, after they are introduced, and after applying GRSN to correct the simulated artifacts. Thus, the performance of GRSN can be evaluated with respect to reducing unwanted variance and improving statistical gene selection performance.

To create a relatively realistic simulated dataset, we used a dataset from a cell culture model with 10 biological control replicates (run on Affymetrix^® ^HG-U133_Plus_2 GeneChips^® ^and processed using the RMA method) to obtain typical background variance (the non-linear artifacts for this dataset were relatively small) [17]. In the first stage of the simulation we randomly partitioned the samples in this dataset into two equal subsets, A and B. We then randomly selected unique subsets of genes and introduced simulated Fold Changes (FC) in the B samples. 1000 genes were set with a FC of 1.5 up, 500 2 fold up, 300 4 fold up, 200 8 fold up; and then 200 were set down 1.5 fold, 200 down 2 fold, and 100 down 4 fold. This gives a total of 2000 up regulated genes with FC in the range of 1.5 to 8 compared to only 500 down regulated genes with FC in the range of -1.5 to -4 so that both the number and degree of up and down regulation is heavily biased in the up direction. In the second stage of the simulation we added random non-linear skew to each sample. The third and final stage of the simulation was to apply GRSN to correct the skew just added. We have repeated this complete simulation, starting with the random partitioning of the original 10 control samples, 100 times (randomly selecting 100 unique permutations from the 252 possible permutations). Figure 4A shows M vs. A plots demonstrating typical skews introduced in a selected sample in two of the 100 different simulations. This figure shows a selected sample compared to the virtual reference sample both before and after the introduction of a simulated skew, and then shows the effect of applying GRSN to correct the simulated skew (compare these plots to Fig. 2).

At each stage of each simulation (after simulated FC is introduced, after simulated skew is added, and after GRSN is applied to correct the simulated skew), the Standard Deviation (SD) within replicates and the average FC between A and B sample subsets is calculated for each gene. A goal of GRSN is to reduce the SD among replicates. As shown in Table 1, the average SD among replicates is highest in the data with simulated skew and is substantially reduced when GRSN correction is applied. The SD after GRSN correction is almost identical to the SD for the original data before simulated skew is introduced (see Table 1). In addition to removing unwanted technical variation, it is important to preserve biologically relevant variation. In this simulation, the biologically relevant variation is the simulated FC introduced in sample set B. Here we calculate the average for all simulated FC ranges up or down across all simulations. In our study, the average FC value stays relatively constant (within 2–3%) at each stage of the simulation (see Table 1), demonstrating that GRSN does not adversely affect the relevant variation (also see Fig. 4C).

**Table 1:** GRSN reduces standard deviation while preserving introduced fold change in a simulated data study.

**Stage of data simulation**	**Avg. SD of replicates**		**Avg. abs FC, samples B vs. A**	
	
	**All Genes**	**Up & Dn Genes**	**Up Genes**	**Dn Genes**
Sim. FC	15.24	24.37	2.67	-1.92
Sim. Skew	35.49	52.55	2.72	-1.90
GRSN correction	14.68	24.97	2.66	-1.92

Average Standard Deviation (SD) among replicates and average Fold Change (FC) are reported for each stage of our simulated data study (see text for description). SD values for each gene are calculated separately for sample set A and sample set B and then averaged across both sample sets and all 100 simulations. FC values are calculated for each gene by taking the average for sample set B and dividing by the average for sample set A then averaging over all 100 simulations. The values reported in each column are (from left to right) 1) the average SD for all genes, 2) the average SD for the up and down regulated genes, 3) the average FC for up regulated genes, and 4) the average FC for down regulated genes. Values reported in the top row are for data with simulated FC only. Values in the middle row are for data with simulated skew added. The bottom row reports values after GRSN correction of the simulated skew.

Next we evaluated the effects of the introduced skews and GRSN correction on statistical gene selection performance in our simulated datasets. Statistically significant genes were selected with eBayes using a FC cutoff of 1.2 and a FDR cutoff of 0.05. We evaluated the numbers of True Positive (TP) (genes with actual simulated FC), False Positive (FP), and False Negative (FN) genes found at each stage of the data simulation for each of the 100 simulations run. Figure 4B shows the results using box plots showing the range of gene selection results across all 100 simulations. The statistical results from the data with simulated artifacts, but no GRSN correction, vary widely from simulation to simulation, resulting in a substantial reduction in identified true positives (middle data set in left plot), and an abundance of false negatives and false positives (middle data sets in middle and right-hand plots). False negatives are more common than false positives due to the random nature of the introduced skew. However, GRSN corrects these issues and the results both before the simulated artifacts and after the simulated artifacts have been corrected with GRSN are very stable (Fig. 4B, compare left and right-hand data sets in each box plot).

We also evaluated the ability of GRSN to preserve the Fold Change (FC) values introduced in the above simulation. We tabulated the average FC for each range over all 100 simulations. This tabulation was done for each stage of the simulation: after simulated FC, after simulated skew, and after GRSN correction. Box plots were used to summarize the results for each FC range and each stage. As seen in Fig. 4C, the variation in FC for each simulated FC range is increased substantially by the simulated skew, but the application of GRSN restores both the mean FC and the variation in FC to values very close to the pre-skew values.

### GRSN corrects non-linear distortions in representative microarray datasets

**Figure 5:**
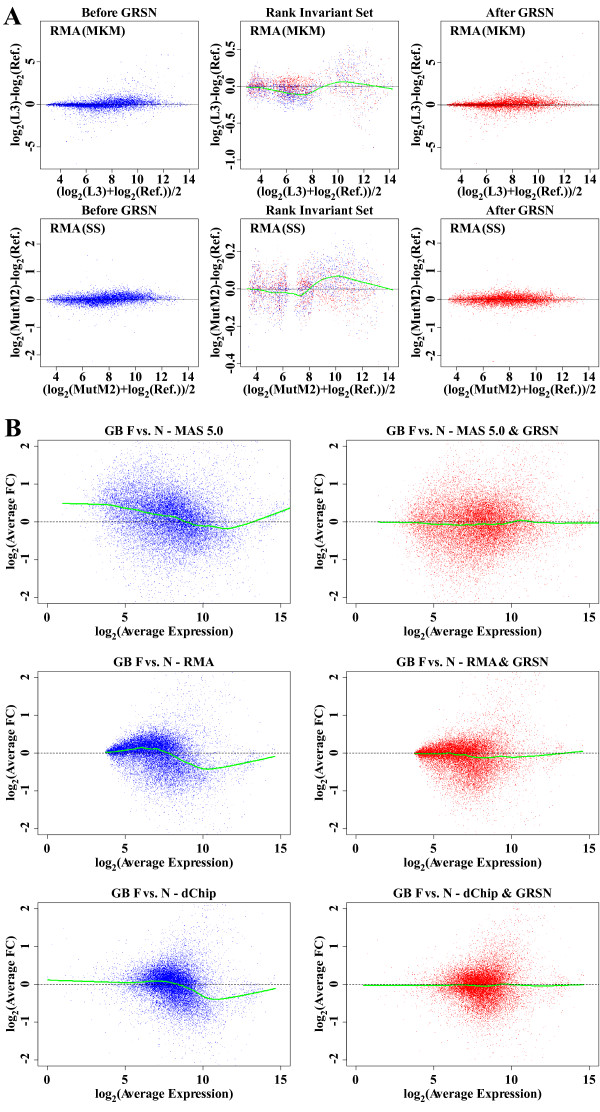
**GRSN corrects non-linear artifacts in representative microarray datasets**. A. GRSN applied to two different microarray datasets. First row – late stage sample L3 from the MKM dataset. Second row – mutant Male sample MutM2 from the SS dataset. Columns 1–3 demonstrate the effect of GRSN on the selected samples as described in figure 2 above. The RMA probe set summary method was used in each. B. GRSN can reduce systematic non-linear artifacts which can affect fold change analysis regardless of pre-processing method. M vs. A plots showing fold change as a function of mean value and plotted on log base 2 scale. Both fold change and mean are calculated using multiple replicates, 14 FA samples and 11 Normal samples from the GB dataset (not just comparing two samples). A lowess smoothed curve is displayed to show the trend of the scatter plots. Three different summary methods are shown: Top row – MAS 5.0, Middle row – RMA, and Bottom row – dChip^®^. The results in the left column are without GRSN applied and the effect of applying GRSN to each of the respective methods is shown in the right column.

We have investigated the application of GRSN on a wide variety of microarray datasets including clinical sample datasets, cell culture datasets with various treatment modalities, and genetic mouse model datasets [18-21] [see Additional file 1]. Two examples are shown in Figure 5A with RMA pre-processing. 1) A mouse model (MKM dataset) of carcinogenesis in cultured clonal keratinocytes [22,23]. Samples were run on the Affymetrix^® ^MOE430A GeneChip^®^. This dataset represents cell culture based experiments with minimal biological variance between replicate samples. 2) A study of limb development in a mouse model (SS dataset) courtesy of Dr. Scott Stadler at OHSU [24]. Samples were run on the Affymetrix^® ^MOE430A GeneChip^®^. This study compares mutant vs. wild type mice with three female and three male replicates for each condition. In both cases we see a reduction in the systematic intensity-dependent artifacts observed in these samples with application of GRSN (Fig. 5A, right-hand column).

When datasets are analyzed for Fold Change between two experimental conditions where each gene's average FC between conditions is plotted versus its average expression for both conditions on M vs. A plots, we also often see non-linear skewing in the data even after averaging replicate samples and regardless of the pre-processing method. This is again likely resulting from the systematic, intensity-dependent artifacts which have no biological significance and it appears at least in some cases to be exacerbated in datasets containing unbalanced numbers of up or down regulated genes. For example, Fig. 5B shows M vs. A plots of the GB dataset comparing 14 Fanconi Anemia samples to 11 normal bone marrow samples before and after applying GRSN. In this example, non-linear distortions are seen without GRSN correction when MAS 5.0 pre-processing is used (top row, left panel), as well as when RMA pre-processing (middle row, left panel), and dChip pre-processing (bottom row, left panel) are used. However, applying GRSN substantially reduces this skew in all cases (Fig. 5B, right-hand panels). Thus, in some datasets, FC assessments can be affected by non-linear artifacts even when averaging multiple, replicate samples and regardless of the probe set summary method used. For each summary method shown, GRSN effectively reduces this skew. The same results are seen with additional datasets [see Additional file 2].

### Reduction of systematic variation by GRSN

**Figure 6:**
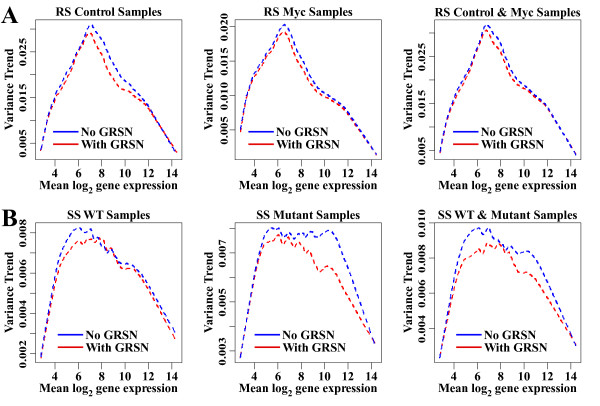
**GRSN reduces average variance in datasets**. Lowess curves are plotted summarizing the variance (for log base 2 scaled data) of all genes among selected sets of replicate samples and whole datasets. The curves show the trend in the variance as a function of expression values. Dashed blue is RMA processed data and dashed red is RMA processed data with GRSN post processing. A. RS dataset showing variance reduction in Control samples, Myc samples, and control and Myc samples combined. B. SS dataset showing, WT samples, mutant samples, and WT and mutant samples combined.

**Figure 7:**
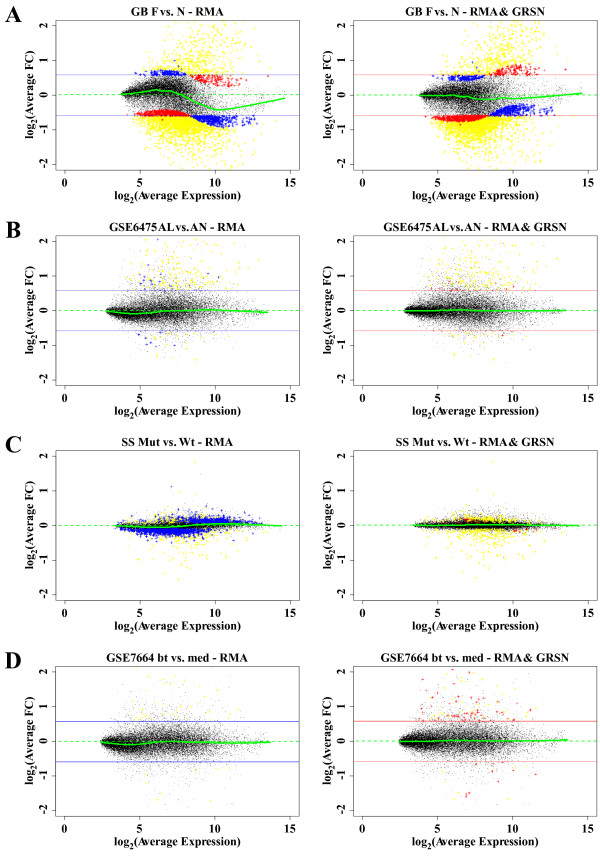
**GRSN impacts gene discovery**. Averaged fold change M vs. A plots as in Figure 5B, but with color coding added to show genes passing fold change and statistical thresholds for significant differential regulation between experimental conditions. Statistical thresholds reported in this figure are for FDR adjusted p-values from standard t-tests. A) GB data comparing 14 Fanconi Anemia samples to 11 Normal samples and plotted using values from RMA method alone (left panel) and using values from RMA with GRSN (right panel). Both plots are color coded to show genes found to be significantly changed (FC of at least 1.5 and FDR of no more than 0.05): genes found only when using RMA alone are in blue, genes found only when using RMA with GRSN are in red, and genes found in both cases are in yellow. The horizontal colored lines show the fold change cutoff applied to the respective summary and normalization methods. B-C) Color coding is modified so that blue genes are shown only in the left panel and red genes are shown only in the right panel. B) GSE6475 data comparing 6 AL (acne lesion) replicates to 6 AN (acne normal) replicates. Samples are plotted as in A. C) SS data comparing 6 mutant samples to 6 wild type (3 male and 3 female for each condition). No FC threshold is applied in this example and the FDR threshold is set to 0.10. D) GSE7664 data comparing 8 bt (treated) to 8 med (untreated) samples plotted as in A.

The goal of GRSN is to reduce systematic non-linear variation in microarray datasets. GRSN is very successful at this task as demonstrated with simulated data. However, there is also random variation in any microarray dataset and this random variation tends to be larger than the systematic variation addressed by GRSN. As a result, applying GRSN will not reduce the variation of all genes and the variation of some genes will actually increase due to the random nature of the non-systematic variation. Still, in most cases, GRSN will reduce the average variation among replicates as shown in Fig. 6. The main benefit seen from this reduction in average variance is in the genes with relatively small random and biological variations. These genes are at the largest risk of becoming false positives due to systematic non-linear artifacts. An example of this is seen in the SS dataset (Fig. 7C).

### Implications of GRSN for gene discovery

An important goal of many microarray-based studies is the identification of genes with statistically significant differential expression between experimental conditions. As seen with the simulated data study and as shown in real data sets in Fig. 5B, the non-linear skew seen in some datasets is likely to significantly impact standard statistical methods for selecting differentially regulated genes. To analyze this we compared statistical results before and after applying GRSN normalization to a number of datasets. We selected significant up and down regulated transcripts that pass a Fold Change threshold of 1.5 and a False Discovery Rate threshold of 0.05 (similar results are obtained using FDR values ranging from 0.01 to 0.20). In Fig. 7A, we use the same M vs. A plots as in Fig. 5B, but add color coding to visualize genes selected as statistically up or down regulated between two sample classes. The "S" shaped skew in the data effects both the calculation of statistical significance and the calculation of fold change. In Fig. 7A, genes from the GB dataset summarized with RMA are color coded based on meeting both a statistical and a fold change cutoff. Transcripts found significant only when GRSN is not applied are indicated in blue, transcripts found significant only when GRSN is applied are indicated in red, and transcripts found significant in both cases are indicated in yellow. The blue horizontal lines indicate the FC threshold applied to select the blue transcripts (left panel) and the red horizontal lines indicate the FC threshold applied to select the red transcripts (right panel). In this case, it can be seen that the skew is pushing large groups of genes in or out of the selected fold change range. In Fig. 7B–D the color coding is modified to show the blue genes only on the left and red genes only on the right and in Fig. 7B there are more blue genes lost with the application of GRSN than there are red genes gained. This result is misleading, because the median p-value for the yellow genes (genes found in both cases) has decreased (improved) from 0.00023 to 0.00021 with the application of GRSN. The reason less genes are found after applying GRSN is due to the FDR adjustment. The p-value required to meet the 0.05 FDR threshold before application of GRSN was 0.0015 while the required p-value after GRSN application was 0.0010. Therefore, the p-value threshold associated with the given FDR value became more stringent after applying GRSN. This is most likely due to the distribution of p-values for genes that did not make the FC cutoff. The cause of this is well illustrated in Fig. 7C with the SS dataset. Here, statistics alone, with the FDR threshold reduced to 0.10 and with no fold change cutoff, are used to select genes. In this case, it appears that there are large groups of genes that are detected as statistically significant due solely to the effect of the "S" shaped skew in the data. In fact, applying GRSN in this case reduces the total number of genes selected from approximately 1,800 to only 171 genes significantly up regulated and 295 genes significantly down regulated (1,344 significant genes were removed and only 20 added). These large numbers of false positive results will cause overly optimistic FDR calculations for all genes and removing these false positive results with the use of GRSN results in fewer genes passing the FDR cutoff even when the actual p-values have improved. In Fig. 7D there are a significant number of red genes added when GRSN is applied and no blue genes lost. In this case, the median p-value for the yellow genes improved significantly from 0.000076 to 0.000042 while the p-value required to meet the FDR threshold changed from 0.00020 to 0.00070. In this case, the FDR threshold became less stringent with the application of GRSN. Presumably the benefit from a decrease in variance among replicates out weighed any bias in the FDR calculation introduced by the removal of false positives (no false positive are shown because they are "masked" by the FC threshold). In summary, systematic distortions in microarray datasets are likely to adversely impact statistical calculations leading to unreliable gene selection results.

### GRSN improves downstream pathway analysis using Gene Set Enrichment Analysis (GSEA)

In addition to examining the effects of GRSN on variance and statistical gene selection we have used the GSEA tool [25] to further analyze the effects of GRSN on downstream microarray data analysis. GSEA looks for the enrichment of known pathways (sets of genes) in the "gene signature" of a particular experiment. Part of the power of GSEA is that it considers the rank and significance of all genes in the gene signature. Therefore, GSEA will benefit both from an increase in True Positives and in a decrease in False Positive gene selection results with the use of GRSN. We have applied GSEA to both the RS and the SS datasets (using the 'R' implementation, version GSEA.1.0.R). The ability of GSEA to detect pathways shown to be relevant in each of these datasets is evaluated both with and without the use of GRSN. As shown in Table 2, both the Normalized Enrichment Score (NES) and the False Discovery Rate (FDR) for these relevant pathways are consistently improved and in some cases, pathways are only detected when GRSN is used. In particular, VEGF is identified as an important player in the SS study [24] but the associated "vegfPathway" is only identified by GSEA when the data is normalized with GRSN. Also, the RS study involves expression of the c-Myc oncoprotein, which is known to induce cell cycle, cell proliferation, cell growth, DNA damage, cell death, and HTERT (see references in Table 2). GSEA identifies all of these pathways to be enriched with c-Myc expression compared to control and as shown in Table 2 all of these pathways are detected at a higher NES and much more significant FDR value when GRSN is used.

**Table 2:** GRSN aids Gene Set Enrichment Analysis (GSEA).

**SS dataset (mutant vs. control)**	**Without GRSN**		**With GRSN**	
**Pathway**	**NES**	**FDR**	**NES**	**FDR**

CR_CYTOSKELETON [28,29]	0.9053	0.6021	1.2209	0.2131
vegfPathway [24]	Not found		1.1346	0.2775
cell_adhesion [30]	Not found		0.9004	0.6332

**RS dataset (Myc vs. control)**	**Without GRSN**		**With GRSN**	

**Pathway**	**NES**	**FDR**	**NES**	**FDR**

Cell_Cycle [31]	1.6516	0.1171	1.7633	0.0240
CR_CELL_CYCLE [31]	1.5565	0.1221	1.6751	0.0488
G1_CELL_CYCLE [31]	1.6902	0.1461	1.6913	0.0398
DNA_DAMAGE_SIGNALLING [32]	1.5355	0.1353	1.6632	0.0490
cell_proliferation [31]	1.6101	0.1161	1.6207	0.0884
cell_cycle_checkpoint [31]	1.4528	0.1941	1.5852	0.1192
PROLIF_GENES [31]	1.5104	0.1539	1.5745	0.1139
cell_growth_and_or_maintenance [33]	1.4340	0.1984	1.5681	0.1099
HTERT_UP [33]	1.4436	0.2064	1.5566	0.1066
cellcyclePathway [31]	1.4618	0.1887	1.4981	0.1357
CR_DEATH [34]	0.9966	0.5629	1.2257	0.3680

GSEA is applied to the SS and RS datasets. Pathways known to be active in these datasets are shown and referenced. For each selected pathway, the Normalized Enrichment Score (NES) and the False Discovery Rate (FDR), as reported by GSEA, are shown. NES and FDR values are shown both for data processed with RMA alone (Without GRSN) and for data processed with RMA followed by GRSN (With GRSN) as indicated.

## Discussion

With any data manipulation there is a risk of adding noise. This is a classic problem with background subtraction where subtracting one noisy signal from another noisy signal can actually double the noise. The goal of GRSN is to remove intensity-dependent, technical variation in the data, but we need to make sure that we do not increase the random noise at the same time. GRSN minimizes this risk in a number of ways. First, by using a global rank-invariant set, we ensure that any noise associated with the selection of the rank-invariant set is applied equally to all samples. This both reduces the risk of adding random variance among replicate samples and reduces the risk of adding bias between sample groups. Second, the iterative method used to select the rank-invariant set ensures that the set is not biased by unbalanced numbers of up or down regulated genes or by unequal degrees of up or down regulation. Third, a single robust virtual reference sample is generated by taking the trimmed mean value (among all samples) of each gene in the global rank-invariant set. This ensures that the linearity of the normalized data is not affected by a few abnormal samples, but is a robust reflection of the dataset as a whole. Forth, the actual intensity-dependent calibration applied to each sample is calculated using a robust lowess smoothing algorithm through many points. Therefore, each sample is compared to the same "representative" virtual reference and the actual calibration is calculated using a rigorous averaging of many reference and comparison data points.

An important distinction between GRSN and other normalization methods is simply when it is applied. We are applying it to the probe set level data after it has been processed and summarized by other methods. This has the advantage that the expression value for a given transcript should more accurately reflect its actual value than will the individual probe values. Other authors have also advocated the application of additional normalization at the probe set level [10]. While we have not investigated applying GRSN to probe level data, we have tried applying the RMA type of quantile normalization to probe set level data. Interestingly, compared to the standard method of applying quantile normalization at the probe level, this yields results more similar to GRSN. We have also investigated substituting lowess normalization for the quantile normalization step in the RMA method (at the probe level) and found it to lead to increased non-linear skew. When GRSN is applied, this increased skew is significantly reduced (data not shown). This suggests that the quantile normalization step is not the cause of the skew and neither is a simple substitution of lowess the solution to the skew.

It is important to note that the typical degree of skewing seen in datasets processed with the RMA method is relatively small in terms of absolute fold change. Therefore, a typical fold change cutoff threshold of 1.5 or 2.0 will mask most of this effect and avoid most false positives. For example, if a fold change cutoff of 2.0 is applied to the data shown in Fig. 7C, all but one of the false positive results reported will be masked, although this is not true for the data in Fig. 7A. However, even when the false positives are masked, they are still affecting the False Discovery Rate calculation for all genes, leading to overly optimistic values. This is probably more of a problem than the errors in the fold change values for the selected genes.

## Conclusion

When microarray data from the Affymetrix^® ^platform is processed using the MAS 5.0 algorithm (still a common practice), it is crucial to check for non-linear artifacts. A simple way to do this is to look at log scale scatter plots comparing pairs of samples. If the trend for any of the scatter plots deviates from the diagonal, additional steps should be taken to normalize the data [10]. The global, rank-invariant set based method (GRSN) presented here is a robust method to address this need. A less obvious and more profound finding is that even when using methods such as RMA or dChip^® ^(with more sophisticated normalization methods built in), it is still important to check for non-linear artifacts. In particular, datasets with unbalanced numbers of up and down regulated genes often have an expression level dependent skew when comparing conditions. GRSN is able to correct this skew without introducing bias. In other words, GRSN does not try to balance up and down gene expression. This skew is not always obvious on a standard log scale scatter plot but can be seen on an M vs. A plot and can adversely impact the calculation of fold change and statistical significance and in some cases can lead to false negative and/or false positive results. Using simulated data where we controlled gene expression and non-linear skew, we have demonstrated how this type of skew can lead to both false negative and false positive gene selection results, and we have shown that GRSN can effectively reduce these artifacts, resulting in much more accurate gene selection performance similar to what was obtained when no skew was present. We also show that GRSN correction of the introduced skew reduces standard deviation among replicates while preserving the magnitude of introduced fold-change. Moreover, we have shown that application of GRSN to real datasets reduces variance among replicates and improves gene pathway enrichment scores for known pathways in datasets.

In summary, GRSN is a robust post-processing normalization step that can correct non-linear systematic distortions in microarray datasets resulting in improved statistical performance, more accurate gene discovery, and enhanced pathway enrichment analysis. GRSN is freely available for non-commercial use [see Availability and requirements section]. This report focuses on data from the Affymetrix^® ^platform, but GRSN should apply equally well to high-density data from other microarray platforms.

## Availability and requirements

This tool is available for free for non-commercial use [see Additional file 3]. Any commercial use requires prior written permission from the author and Oregon Health and Science University and may require licensing fees. An implementation based on the "R" statistical computing language is provided with source code included. The scripts have been developed and tested using Windows XP^®^, but should work on other operating systems which are supported by the "R" statistical platform, including MacOS and Linux. Multiple "R" versions have been tested, including version 2.4.0. Future updates and additional information will be provided at: Sears Lab when available.

## Methods

### Software

All statistical analysis, method implementation, and graphing for figures, was performed using the open-source programming language R, Versions 2.0.1 – 2.4.0 [26], and Bioconductor packages . Affymetrix^® ^CEL files were processed using the MAS 5.0 and RMA algorithms implemented in the BioConductor package: affy Version 1.5.8 [27] (based on the R programming language) and by dChip^® ^2006 (Build date: Feb. 16, 2006). Gene Set Enrichment Analysis (GSEA) was performed using the author's R language implementation version GSEA.1.0.R [25].

### GRSN Implementation

At the heart of the GRSN method is the selection of a global rank-invariant set. Microarray data is passed to GRSN as a matrix of expression values with rows representing transcripts (probe sets) and columns representing samples. The Affymetrix^® ^control transcripts are excluded. Non log scaled data is expected. If necessary, a fixed value is added to all matrix entries to ensure that no entry is less than 0.25. This is to ensure that when the data is log base 2 (log2) scaled, no values will be less than negative 2.0. The data is then log2 scaled. Next, a rank matrix of the same dimension as the input matrix is calculated. For each column of the input matrix, the rank (order in a sorted list) of each transcript in that column is calculated and used to populate the rank matrix. The variance of each row of the rank matrix is calculated and the rows are then ranked based on their variance. The rows with the highest variance are discarded. A subset of the original log2 scaled matrix is produced, containing only the rows corresponding to the remaining rows in the rank matrix. Using this new matrix, another rank matrix is calculated. This process is repeated in an iterative manner four (4) times. (The number of rows to discard per iteration is calculated by subtracting the selected size (default is 5000) for the Global Rank-invariant Set (GRiS) from the original row count and dividing the result by four.) The remaining transcripts are considered the GRiS. Next, reference values for each of the transcripts in the GRiS are obtained by calculating the trimmed mean from all samples for each of the respective transcripts. A calibration curve is then calculated for each sample by comparing the GRiS values from the given sample to the GRiS reference values using an M vs. A plot. The plotted points representing the GRiS should be centered about the horizontal line where M = 0. The calibration curve is obtained by drawing a smoothed line through these points using lowess [13] as implemented in R (the default smoother span used with GSRN is 0.25, but this value may be adjusted if needed). The vertical displacement between the smoothed calibration curve and the horizontal line (M = 0) is used as an intensity dependant adjustment to be applied to all transcripts in the given sample. This adjustment is subtracted from the log2 scaled data (this is equivalent to applying an intensity dependant scaling factor to non log scaled data). It should be noted that the lowess smoothing and the calculation of the intensity dependant adjustment values are done on the data after it has been transformed for the M vs. A plot. After the intensity dependant adjustment is applied, the M vs. A transformation is reversed. Our implementation of GRSN as an "R" script with directions is available [see Additional file 3].

### GRSN Graphical Visualization

Quality control graphs are plotted for each sample as it is normalized. For each sample, three M vs. A scatter plots are generated: The first is pre GRSN, and compares the given sample to the virtual reference sample (a pseudo sample with each transcript set to the trimmed mean value for that transcript from all samples in a dataset). The second shows the GRiS transcripts only, both before and after adjustment, and includes the lowess smoothed calibration curve. The third shows the given sample, after adjustment, compared to the virtual reference sample.

### Data simulation study

GRSN was evaluated using simulated data produced by adding simulated gene expression and simulated non-linear artifacts (skew) to a cell culture dataset containing 10 replicated controls [14]. The 10 samples were first processed using the RMA method. Next, all possible permutations for dividing these 10 replicate samples into two groups of 5 (set A and set B) were calculated. Out of the 252 possible permutations, we randomly selected 100, thus ensuring that we had 100 randomly selected, yet distinct partitions of the original data. For each of these 100 distinct partitions (hereafter called a simulation), we added simulated Fold Change (FC) by applying a multiplication factor to selected transcripts (genes) in all samples in set B. For each simulation, we randomly selected 2500 genes. The first 1000 (out of the 2500 selected) were multiplied by +1.5 to simulate a FC of 1.5 up. A factor of +2 was applied to the next 500, a factor of +4 to the next 300, a factor of +8 to the next 200, a factor of 2/3 to the next 200, a factor of 1/2 to the next 200, and a factor of 1/4 to the final 100 genes. Thus, up regulation with FC in the range of 1.5 to 8 was simulated for 2000 genes and down regulation with FC in the range of -1.5 to -4 was simulated in 500 genes. The next step in each simulation was to add distinct, randomly generated skew to each sample. This was done by randomly generating curves similar to the calibration curves produced by GRSN when normalizing RMA processed data and using these as "calibration curves" to add intensity-dependent skew to the simulated samples. The final step in each simulation was to apply GRSN to correct the introduced skew. The simulated data was analyzed at each of the three stages in the simulation process just described, 1) after simulated FC was introduced, 2) after simulated skew was added, and 3) after GRSN was applied to correct the simulated skew. The results of these analyses were tabulated and/or averaged over all 100 simulations and reported in the results section.

## Authors' contributions

CP developed the GRSN application, wrote all of the software, created the application web site, prepared and analyzed all of the data presented in this study, and wrote the manuscript. RS supported and helped CP with all of the data analysis in this study, figure preparation, and with writing of the manuscript. GB and MK-M provided unpublished data for analysis and scientific discussion of the GRSN algorithm.

## Supplementary Material

Additional file 1 **Additional examples of GRSN.** Scatter plots showing the results for selected samples when applying GRSN to various datasets. Click here for file

Additional file 2 **Additional examples of a global skew corrected by GRSN.** Scatter plots showing global skew between experimental conditions before and after correction with GRSN. Click here for file

Additional file 3 **Mini website with code and instructions for GRSN method.** This file contains a zipped directory structure to provide a mini website with code and directions which provide an implementation of the GRSN method using the open source “R” environment. Click here for file
