# Supplementary material for: Global rank-invariant set normalization (GRSN) to reduce systematic distortions in microarray data
Source: BMC Bioinformatics. 2008 Dec 4;9:520. doi: 10.1186/1471-2105-9-520 (PMC2644708; doi:10.1186/1471-2105-9-520)
Supplement: Additional file 1 — Additional examples of GRSN. Scatter plots showing the results for selected samples when applying GRSN to various datasets. [file 1471-2105-9-520-S1.pdf]

Supplementary Figure 1. Additional examples showing the GRSN method applied to RMA processed data (see figures on following pages).

Datasets included in this figure are: **GB** dataset – a study of the inherited disease, Fanconi Anemia, using patient bone marrow samples run on the Affymetrix® HG-U133A GeneChip®. This data includes 11 primary bone marrow samples from Normal patients (N1 – N11) and 14 bone marrow samples from patients with Fanconi Anemia (F1 – F6 & F8 – F15) whose bone marrows were hypoplastic but not leukemic. This dataset represents the more heterogeneous data typical of a clinical study. **SS** dataset – a study of limb development in a mouse model courtesy of Dr. Scott Stadler at OHSU [24]. This study compares mutant vs. wild type mice with three female and three male replicates for each condition. **GSE6475** dataset – a study of acne lesions in human patients [18]. This dataset includes paired samples from acne lesions and normal skin from 6 human subjects as well as normal skin samples from 6 human patients without acne. Affymetrix® HG-U133A 2.0 arrays were used and data was contributed to the GEO database under accession number GSE6475. **GSE6802** dataset – a profiling of stimulated bronchial epithelial cells [19]. Control and stimulation conditions were done in triplicate with one exception, which was done in duplicate. Affymetrix® HG-U133A 2.0 arrays were used and the data was contributed to the GEO database under accession number GSE6802. **GSE4561** dataset – a study of Nox1 inhibition in human colon cancer contributed to the GEO database under accession GSE4561 (submission date: Mar 27, 2006 and Contact name: Agnes Juhasz). In this study, conditions were replicated three or four times using Affymetrix® HG-U133A 2.0 arrays. This dataset is representative of microarray data with small non-linear artifacts. **GSE7664** dataset – a study of human cell response to benzene metabolites contributed under GEO accession GSE7664 [20]. This dataset contains 8 treated and 8 untreated samples, was produced using Affymetrix® HG-U133A 2.0 arrays, and is representative of microarray data with moderate non-linear artifacts. **GSE1692** dataset – a study of T98G growth arrest contributed under GEO accession GSE1692 [21]. Each of 3 conditions was replicated in triplicate using Affymetrix® HG-U133A 2.0 arrays. This dataset is representative of microarray data with small to moderate non-linear artifacts. **RS** dataset – an unpublished dataset from the Sears lab. An investigation of Myc stabilization in a mouse model using Affymetrix® Mouse430_2 arrays. This data is included as an example of the current, higher density generation of GeneChips®. A subset of this data, including 5 stable T58A mutant (TA) and 4 Control samples, is shown here.

Column 1 shows M vs. A plots comparing one selected sample to the reference sample created by taking the median expression value of each probe set in that dataset. Column 2 shows the global rank-invariant set of 5,000 probe sets before GRSN normalization in blue and after normalization in red. The smoothed curve through the rank-invariant set is shown in green. This is the calibration curve used to normalize the selected sample. Note the expanded scale of the y-axis for the graphs in this column. Column 3 shows all probe sets after GRSN normalization. **A**. Sample F4 from the GB data set. **B**. Sample AN3 from the GSE6475 data set. **C**. Replicate sample S1 from the GSE6802 data set. **D**. Replicate sample S2 from the GSE6802 data set, the previous three samples were chosen for their large non-linear artifacts, while this sample was chosen as a comparison to the previous sample. Note that the artifact seen for this sample is smaller but in the opposite direction of the artifact for the previous sample which is a biological replicate. **E**. Sample HtNox3 from the

GSE4561 data set. **F**. Sample bt7 from the GSE7664 data set. This sample was chosen as an example of minimal non-linear artifacts. All other samples from this data set have larger artifacts. **G**. Sample CI3 from the GSE1692 data set. **H**. Sample TA1 from the RS data set. This sample was chosen as a typical example from a current generation Affymetrix® GeneChip®.

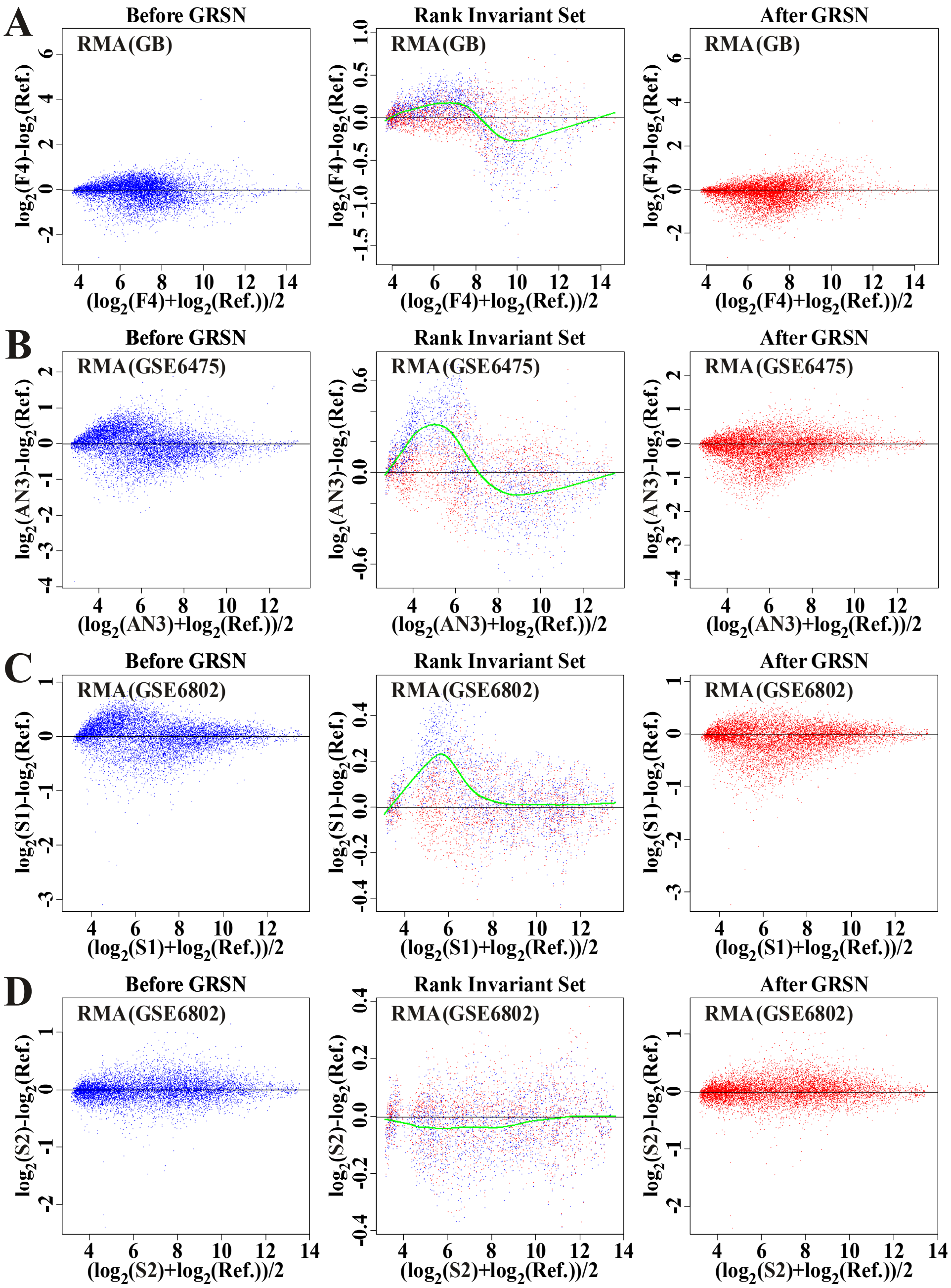

Supplementary Figure 1 A-D

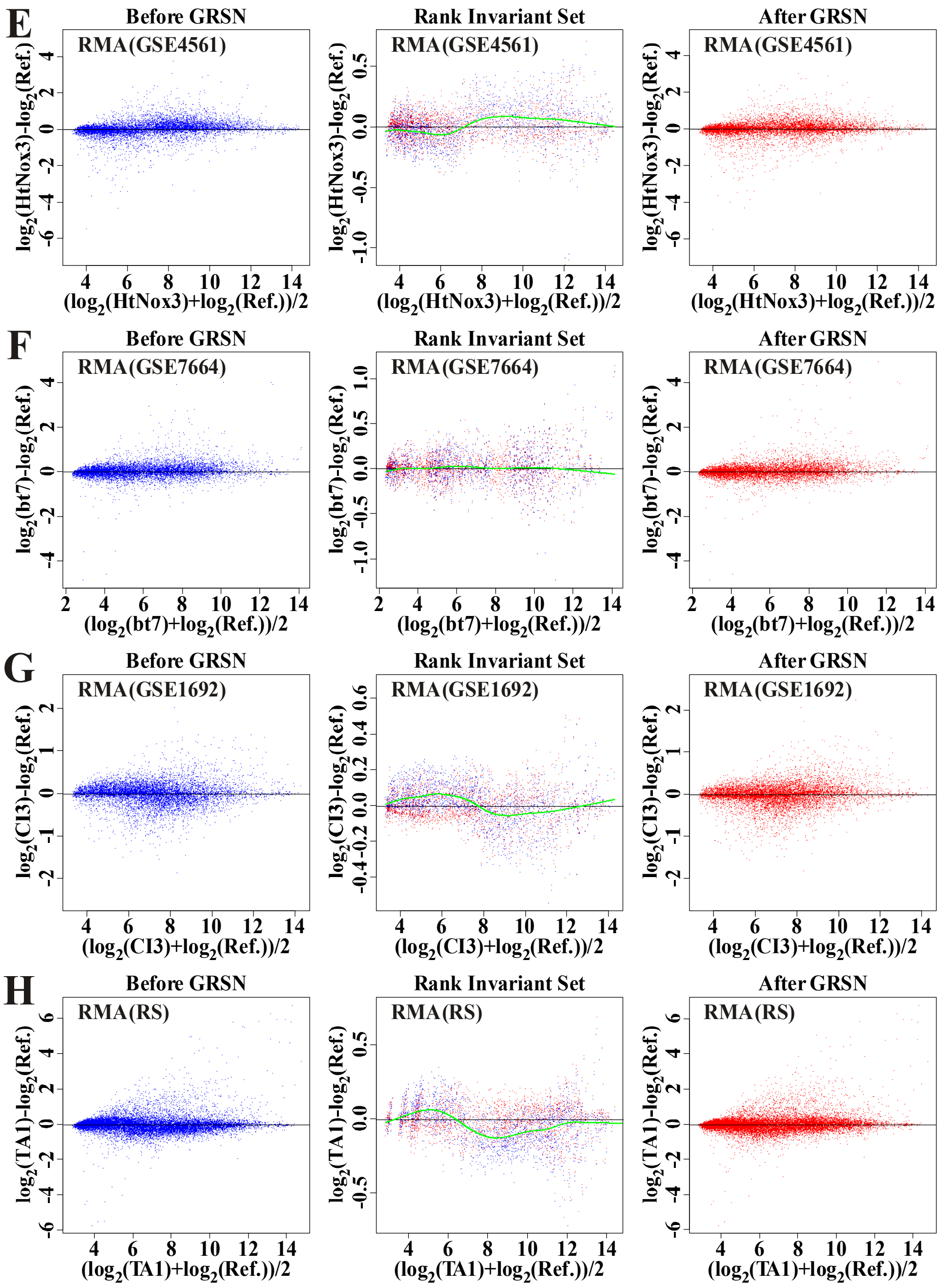

Supplementary Figure 1 E-H
