# Supplementary material for: Global rank-invariant set normalization (GRSN) to reduce systematic distortions in microarray data
Source: BMC Bioinformatics. 2008 Dec 4;9:520. doi: 10.1186/1471-2105-9-520 (PMC2644708; doi:10.1186/1471-2105-9-520)
Supplement: Additional file 2 — Additional examples of a global skew corrected by GRSN. Scatter plots showing global skew between experimental conditions before and after correction with GRSN. [file 1471-2105-9-520-S2.pdf]

Supplementary Figure 2. Additional examples of artifacts seen when comparing multiple, replicate samples between experimental conditions (also see next page).

See Supp. Fig. 1 for descriptions of datasets shown in this figure. M vs. A plots showing fold change as a function of mean value and plotted on log base 2 scale. Both fold change and mean are calculated using replicate samples from each of two experimental conditions (not just comparing two samples). A lowess smoothed curve is displayed to show the trend of the scatter plots. RMA processed data is shown. The datasets and conditions compared are: **A**. GSE6475 AL vs. AN, **B**. GSE6802 R vs. C, **C**. GSE4561 HtS vs. HtNox, **D**. GSE7664 bt vs. med, **E**. GSE1692 ASNY vs. GO, and **F**. RS TA vs. C. The results in the left column are without GRSN applied and the effect of applying GRSN is shown in the right column. These examples demonstrate systematic non-linear artifacts between experimental conditions even after averaging replicate samples. These artifacts will bias the results from statistical analysis of differential gene regulation. The degree of artifact seen here is typically less then what was shown for the GB data set, however some degree of artifact is seen in most if not all data sets.

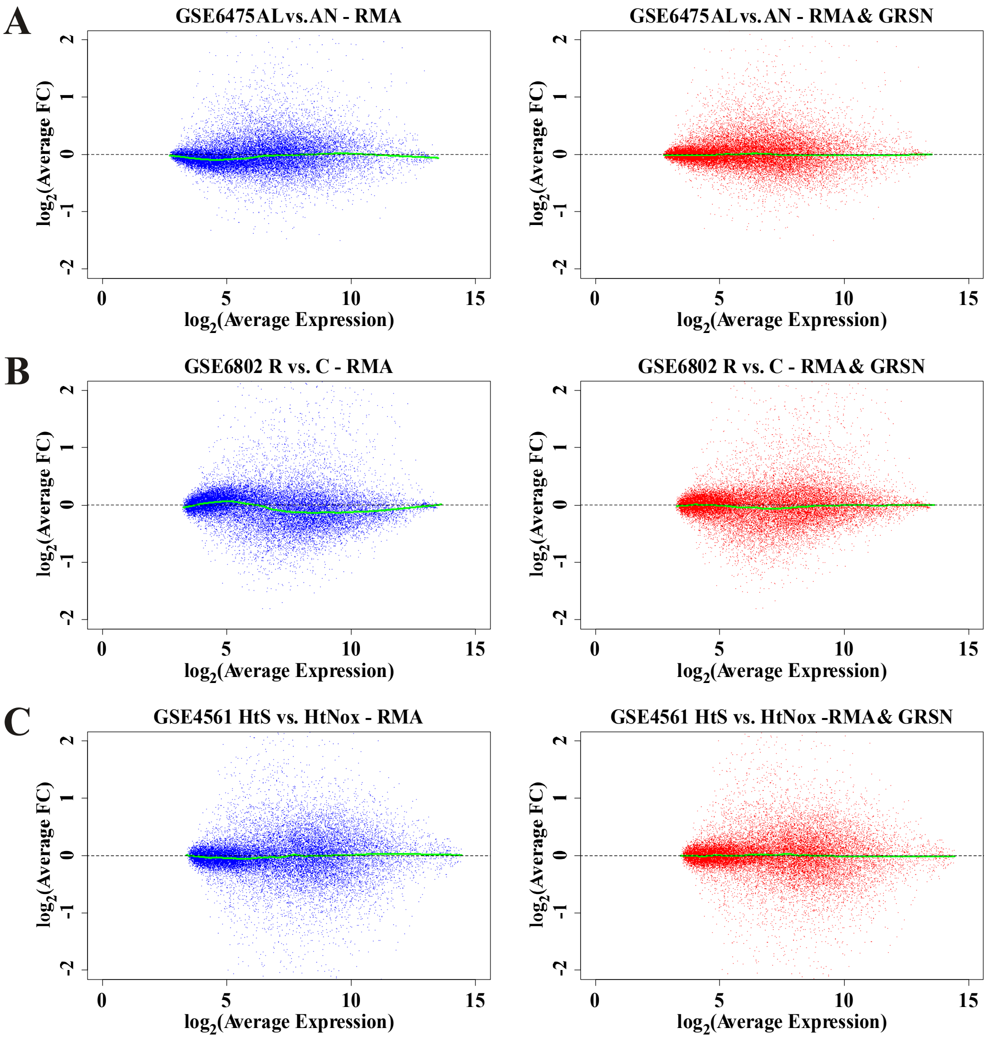

Supplementary Figure 2 A-C

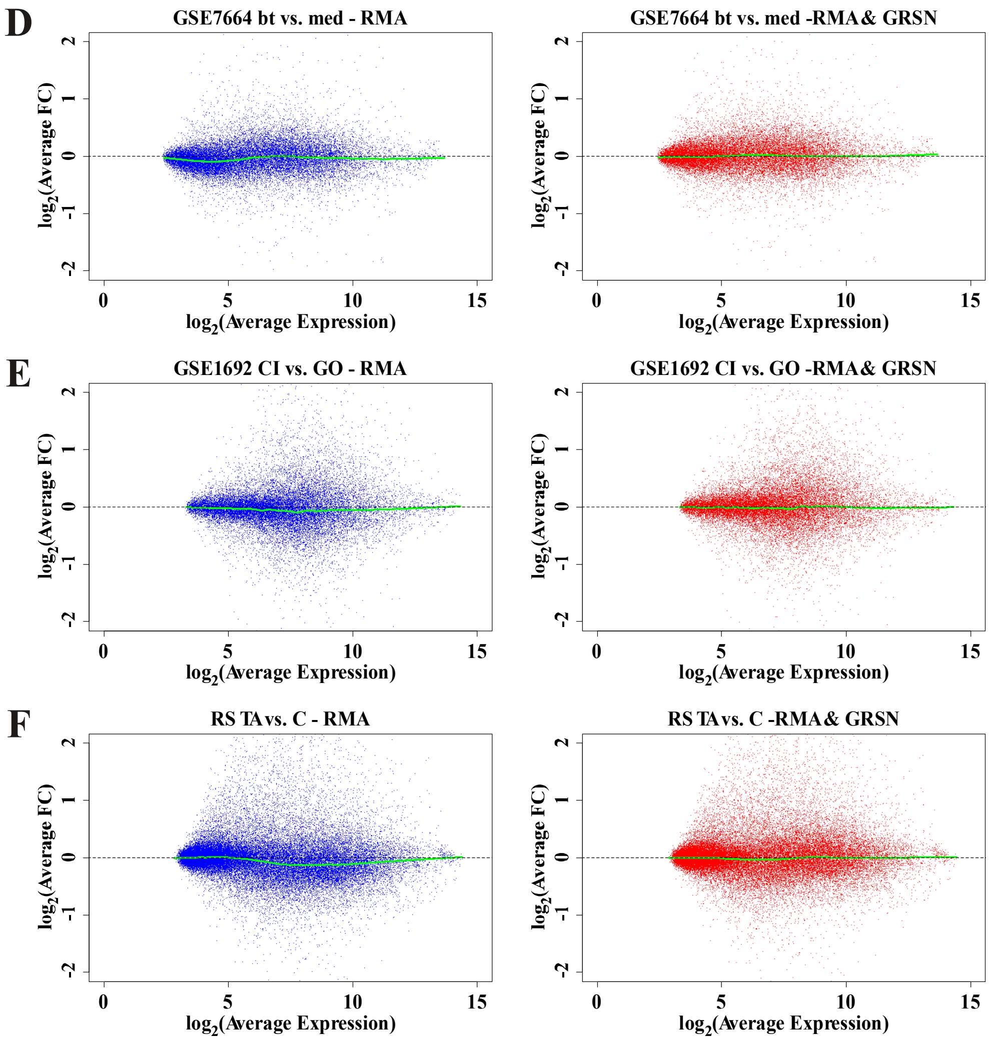

Supplementary Figure 2 D-F
