# Supplementary material for: Global rank-invariant set normalization (GRSN) to reduce systematic distortions in microarray data
Source: BMC Bioinformatics. 2008 Dec 4;9:520. doi: 10.1186/1471-2105-9-520 (PMC2644708; doi:10.1186/1471-2105-9-520)
Supplement: Additional file 3 — Mini website with code and instructions for GRSN method. This file contains a zipped directory structure to provide a mini website with code and directions which provide an implementation of the GRSN method using the open source “R” environment. [file 1471-2105-9-520-S3.zip › GRSNMiniSite/index.html]

Global Rank-invariant Set Normalization (GRSN)


# Global Rank-invariant Set Normalization (GRSN)


## by Carl Pelz


© 2004-2008   

|  |
| --- |
| Introduction:  The purpose of this web page is to provide scientists and biostatiscians access to an implementation of our GRSN normalization method. This method and implementation are provided free of charge for non-commercial academic use. Any commercial use requires prior written permission from the author and Oregon Health and Science University and may require licensing fees. Global Rank-invariant Set Normalization (GRSN) is designed to reduce specific types of unwanted technical variation in microarray datasets. The implementation provided here is in the form of an R script and is designed to be used with Affymetrix® GeneChip® data that has been processed using the affy package which is available at the R website. GRSN Script |
| Getting Started:  - The first step is to install R, a free software environment for   statistical computing and graphics. R is available for a wide variety   of UNIX platforms as well as Windows and MacOS. Please see:   www.r-project.org   for directions on obtaining R. - Once R is installed, you will need to install the affy package from   the Bioconductor repository. The details will depend on the operating   system that you are using. In the Windows environment, you would start   the RGui application and use the "Packages" -> "Select repositories..."   menu to select the "BioC software" repository. Next, you would use the   "Packages" -> "Install package(s)..." menu to select and install the   "affy" package. Note that you may also be asked to select an appropriate   "mirror" near your location. - Next, you will need to make sure all your CEL files from your   Affymetrix® microarray experiment are in a single folder. You will   need to start an R session and make sure that your working directory   is set to the folder with your CEL files. You can use the "File" ->   "Change dir..." menu from within your R session, or you can use the setwd()   function from the R command line. - Finally, you can use the R script that we provide   HERE   to process your data using the RMA method provided within the affy   package AND apply the additional normalization provided by the GRSN method.   Just cut and paste this complete script into your R session. - The GRSN method will generate files named "TestGRSN##.png"   where the ## are sequential numbers for your CEL files (samples). These files   will be placed in the same folder as your CEL files. These are images showing   M vs. A plots of each sample before and after application of GRSN as well as a   M vs. A plot showing the Global Rank-invariant Set GRiS used. Inspection of these   images will tell you how much effect GRSN had on your particular dataset and give   you an indecation of the quality of the GRiS selected. See sample here |

References  
  
**R environment:**
  
R Development Core Team (2006). R: A language and environment for
statistical computing. R Foundation for Statistical Computing,
Vienna, Austria. ISBN 3-900051-07-0, URL http://www.R-project.org.
  
  
**affy package:**
  
Rafael A. Irizarry, Laurent Gautier, Benjamin Milo Bolstad, and
Crispin Miller with contributions from Magnus Astrand
, Leslie M. Cope, Robert Gentleman,
Jeff Gentry, Conrad Halling, Wolfgang Huber, James MacDonald,
Benjamin I. P. Rubinstein, Christopher Workman and John Zhang (2006).
affy: Methods for Affymetrix Oligonucleotide Arrays. R package
version 1.12.2.

---

Sears lab   
**Last updated:** July 12th, 2008
