# Supplementary material for: Global rank-invariant set normalization (GRSN) to reduce systematic distortions in microarray data
Source: BMC Bioinformatics. 2008 Dec 4;9:520. doi: 10.1186/1471-2105-9-520 (PMC2644708; doi:10.1186/1471-2105-9-520)
Supplement: Additional file 3 — Mini website with code and instructions for GRSN method. This file contains a zipped directory structure to provide a mini website with code and directions which provide an implementation of the GRSN method using the open source “R” environment. [file 1471-2105-9-520-S3.zip › GRSNMiniSite/SampleGRSNimage.html]

Arial Photo


BACK
